# Isolation and characterisation of exosomes from Chinese hamster ovary (CHO) cells

**DOI:** 10.1007/s10529-023-03353-3

**Published:** 2023-01-28

**Authors:** Eleftheria Skrika-Alexopoulos, C Mark Smales

**Affiliations:** Industrial Biotechnology Centre and School of Biosciences, University of Kent, Canterbury, CT2 7NJ UK

**Keywords:** Exosomes, Chinese hamster ovary (CHO) cells, Characterisation

## Abstract

Exosomes have previously been isolated from Chinese hamster ovary (CHO) cells and their anti-apoptotic properties reported. However, to further facilitate the study of CHO cell derived exosomes and allow their comparison across studies, it is necessary to characterise and define such exosomes using at least three criteria that can act as a reference for the generation of CHO cell produced exosomes. Here we report on the isolation of exosomes from CHO cells, an industrially relevant and widely used cell host for biopharmaceutical protein production, during the exponential and stationary phase of growth during batch culture using a Total Exosome Isolation (TEI) method. The resulting vesicles were characterized and visualized using a diverse range of techniques including Dynamic Light Scattering (DLS), Zeta potential, Electron Microscopy and immunoblotting, and their protein and RNA content determined. We also generated the lipid fingerprint of isolated exosomes using MALDI-ToF mass spectroscopy. We confirmed the presence of nano sized extracellular vesicles from CHO cells and their subsequent characterization revealed details of their size, homogeneity, surface charge, protein and RNA content. The lipid content of exosomes was also found to differ between exosomes isolated on different days of batch culture. This analysis provides a profile and characterisation of CHO cell exosomes to aid future studies on exosomes from CHO cells and improving the manufacturing of exosomes for biotherapeutic application.

## Introduction

Exosomes are defined as lipid bilayer membrane-enclosed extracellular vesicles of around 30–150 nm in diameter that are formed as endosomal multivesicular compartments/bodies (MVB) before fusing with the plasma membrane and being secreted into the extracellular milieu. Exosomes belong to a larger group of extracellular vesicles (EV) that includes ectosomes and apoptotic bodies (Lobb et al. 2015; Li et al. 2017; Lee et al. 2012). Their role in intercellular communication in both physiological as well as pathological states has led to a growing interest in their isolation and characterisation from various sources in order to understand their biology, as potential biomarkers and as therapeutic agents (Bin Wang et al. 2019). Indeed, exosomes can cross the blood–brain barrier (Saeedi et al. 2019) and hence they have a potential wide scope as therapeutic agents.

The isolation, purification, characterisation and detection of exosomes remains technically challenging and various methodologies have been employed to isolate and define and/or characterise exosomes (Li et al. 2017). Difficulties in isolating and defining an exosome preparation come from their small and overlapping sizes, their heterogeneous morphology and composition, and co-isolation with other molecules and complexes. As a result, researchers in the field have defined a number of criteria that represent the minimal number of methodologies that should be employed to characterise these vesicles in any study (Lotvall et al. 2014). In the present study, the term ‘exosomes’ is used to refer to exosome enriched preparations (Tang et al. 2017).

As exosomes have a wide range of potential applications (Dyball et al. 2022), a further challenge in their application is the manufacturing at an appropriate scale from a system that is suitable for generating material that can be used in humans. Exosomes have traditionally been investigated and sourced from a number of different cell types (Hadizadeh et al. 2022), however defined, scalable and industry wide adopted manufacturing processes for generating exosomes from different cell sources are yet to be established. One system that is widely used for the generation of biotherapeutic proteins destined for use in humans (Dyball et al. 2022; Feary et al. 2017; Mead et al. 2015; Povey et al. 2014) and that could act as a system for the production of exosomes is the Chinese hamster ovary (CHO) cell line. Indeed, exosomes isolated from CHO cells have recently been shown to exhibit anti-apoptotic effects (Han et al. 2018). Further, a number of recent studies have described the potential of CHO cells as a scalable source of therapeutic exosomes and the desperate need for further research into CHO cell derived exosomes and their characterisation (Belliveau et al. 2022; Busch et al. 2022; Keysberg et al. 2021). Collectively these studies evidence a growing interest in CHO cell exosomes and the need for methods to quantify and study these.

However, to facilitate the study of CHO cell derived exosomes and allow comparison across studies it is necessary to isolate and characterise/define CHO cell derived exosomes using at least the minimal criteria outlined by Lotvall et al. (2014) that can then act as a reference for the generation of CHO cell exosomes. Some recent excellent studies have started to define the characteristics of CHO cell based exosomes including those that have defined the mRNA transcripts and microRNAs that are present in CHO cell derived exosomes (Busch et al. 2022), reports of 1395 proteins identified to be in CHO cell generated exosomes alongside various RNA species (Keysberg et al. 2021) and descriptions of how EVs allow exchange of protein and RNA content between cells (Belliveau et al. 2022).

Here we report on the isolation of exosomes from CHO cells, an industrially relevant and widely used host for biopharmaceutical protein production (Budge et al. 2020a; Vito et al. 2020). We have utilized a commercially available polymer based precipitation (PBP) technique, Total Exosome Isolation (TEI), to isolate and enrich extracellular exosome vesicles from batch culture. Such commercial isolation methods have grown in popularity in recent years as they are less time-consuming, potentially more reproducible and ‘user friendly’, and achieve high yields of vesicles exhibiting high purity of small RNAs as well as preserving their biological activity compared to traditional centrifugation based methods that can differ from user to user (Tang et al. 2017; Niu et al. 2017; Peterson et al. 2015; Ferguson et al. 2018; Han et al. 2018). Using this approach, we have isolated exosomes from CHO–S cells, one of the main CHO cell hosts that is industrially relevant, grown in suspension during the exponential and stationary phases of growth of batch culture to define CHO cell exosome fingerprints. We then applied a combination of methodologies to detect and characterise the isolated vesicles, including Dynamic Light Scattering (DLS) and Zeta Potential measurements, electron microscopy (EM), and protein (via western analysis of exosome markers), RNA and lipid profiling. The resulting analyses define CHO cell exosome properties that can be used as a reference for CHO cell exosome studies by others from two distinct growth phases of batch-culture.

## Materials and methods

### Cell culture

CHO–S cells and CD–CHO medium were purchased from ThermoFisher Scientific. Cells were grown in CD–CHO media to which 8 mM L-glutamine was added with cultures routinely been grown in 20 ml batch-culture mode in Erlenmeyer flasks at 37 °C with shaking at 120 rpm. Cells were passaged every 3–5 days with seeding at 0.2 × 10^6^ viable cells/ml. For batch-cultures from which exosome preparations were prepared, cells were seeded at 0.2 × 10^6^ viable cells/ml in 20 ml cultures and then the supernatant harvested on days 3 and 5 of culture, at what we considered exponential and stationary phase as previously determined (Vito et al. 2018), respectively, from two independent cultures. Three samples of 1.0 × 10^6^ cell pellets and five samples of 3 × 10^6^ cell pellets were also aliquoted for cellular lipid and protein analysis respectively, at both harvest days. Cells were pelleted by centrifugation for 5 min at 2,000×g and stored at − 80 °C. All of the remaining culture supernatant was used for the isolation of exosomes as described below.

### Exosome preparation by polymer-based precipitation (PBP)

Exosomes were isolated from the culture media using the commercially available Total Exosome Isolation (TEI) system (lot #0,049,003, Invitrogen, Life Technologies) according to the manufacturer’s guidelines. The cell culture media was centrifuged at 2000×*g* for 30 min to remove cells and any debris; subsequently the supernatant was carefully removed to a new tube and 0.5 volumes of TEI reagent added, thoroughly mixed and incubated at 4 °C overnight. Samples were then centrifuged at 10,000×*g* at 4 °C for 1 h, and then after discarding the supernatant, the exosome enriched pellets re-suspended in 75 μl of PBS buffer. The samples were then stored at − 20 °C until required for subsequent analysis.

### Characterisation of exosomes

#### Dynamic light scattering

The particle size distribution in exosome preparations were determined using an Anton-Paar Lightsizer 500 Particle Analyzer. Dynamic light scattering measures the dynamic fluctuations in the intensity of scattered light due to the Brownian motion of the particles in solution, (laser light of 685 nm with detection angles at 15, 90, 175 degrees); smaller particles move faster than larger ones. There are three commonly used types of distributions when the size of particles is determined; (1) the number distribution which reports the number of particles in different size “bins”; (2) the volume distribution which reports the total volume of particles in different size categories; (3) the intensity distribution which reports the light scattered by particles of different size. In this study, all estimations of exosome size distribution used the number distribution as opposed to the intensity distribution commonly used in the literature (Kesimer et al. 2015) because this better describes the actual *number* of vesicles of each size in the sample and smaller sized particles are more appropriately represented (zetasizer nano technical note mrk1357-01). For analysis, 75 μl aliquots of exosome preparations previously stored at − 20 °C were diluted with 925 μl of PBS (to give 1 ml) and transferred into disposable cuvettes for DLS measurements. Each sample was measured five times in both the DLS and Zeta potential experiments as outlined below.

#### Zeta potential

Zeta potential, measured by electrophoretic light scattering (ELS), measures the speed of the particle in an electrophoretic field. Zeta potential is an indicator of the stability in colloidal dispersions (absolute value) and a measure of the charge on the surface of the vesicle (+ or − sign). Zeta potential was estimated on an Anton- Paar Lightsizer 500 Particle Analyzer.

#### Electron microscopy

For electron microscopy analysis, exosome preparations were diluted 1:10 in PBS buffer and subsequently fixed in an equal volume of 4% (w/v) paraformaldehyde and 1% (v/v) glutaraldehyde for 5 min. Samples were then placed on formvar-carbon coated 600 mesh copper grids and dried at room temperature for 5 min. The samples were subsequently contrasted in a solution of uranyl acetate (2% aqueous solution) and viewed under an electron microscope (Jeol1230 TEM) with an accelerating voltage of 80 kV coupled to a camera (Gatan One View) (Niu et al. 2017).

#### Determination of protein content in samples by Bradford analysis

In order to quantify the protein content in CHO cell pellets and exosome preparations from days 3 and 5 of culture, a Bradford assay was performed on cell lysates, as well as 75 μl aliquots, each originating from 1 ml of culture, of exosome lysates and non-lysed exosomes (Bradford 1976). Lysates were prepared by adding lysis buffer previously described to the cell or exosome preparation (Roobol et al. 2015).

#### SDS-PAGE and western blotting

Western blotting was performed on both lysed and non-lysed exosome preparations, and cell lysates from days 3 and 5 of batch culture. All samples were prepared either in reducing (containing β-mercaptoethanol) or non-reducing Laemmli sample buffer, boiled for 5 min at 95 °C and subsequently resolved by 12% SDS-PAGE as previously described (Zhang et al. 2016). Approximately 5 g of each protein sample was loaded. The following primary antibodies were used for western blotting of known exosome markers; anti-CD63 (Santa Cruz, sc-5275), anti-CD81 (Santa Cruz, sc-166029 and sc-23962), and anti-TSG101 (Santa Cruz, sc-136111).

#### RNA isolation and analysis

Total RNA was extracted from 3 exosome samples from Day 3 and Day 5 of harvest. The Total Exosome RNA (TER) and Protein Isolation Kit (Invitrogen, ref 4,478,545) was used to extract the RNA according to the manufacturer’s instructions. 30–35 l RNA was eluted from each exosome sample that originated from 1 ml of culture. The total amount of RNA recovered was then estimated using a NanoDrop ND-1000 spectrophotometer.

#### Lipid Analysis

Lipids were extracted from (a) total cell pellets (1 × 10^6^ viable cells containing 298.9 g of protein when harvested at day 3 and 477.2 g of protein when harvested at day 5) that had been stored at − 80 °C, and (b) from the pooled exosomes isolated from 7 × 1 ml of culture (i.e. we took 7 × 75 μl exosome aliquots each isolated from 1 ml of culture for each time point and then pooled these together) essentially as previously described (Budge et al. 2020a, 2020b). The lipids were then extracted from these samples by adding a chloroform:methanol (2:1) solution (3 ml) to the cells/exosome preparations, which was then left for 20 min before adding Milli-Q grade H_2_O (500  l) and then centrifuging the mixture for 5 min at 1500 g. The chloroform (lower) lipid-containing phase was then removed, dried under N_2_ and stored at − 80 °C (Llorente et al. 2013). The samples were subsequently analysed using matrix-assisted laser desorption ionisation time-of-flight (MALDI-ToF) mass spectrometry (MS) as described by Fuchs et al. (2010). Each sample (1 μl) was spotted onto a MTP 384 target ground steel T followed by matrix (1 μl as outlined below) and the samples then analysed in manual mode (both + ve and -ve ion mode) using an ultraflextreme Bruker MALDI-ToF mass spectrometer. The lipid profiles of the cells at days 3 and 5 of culture, and of the isolated exosomes from day 3, were also analysed in the automatic mode using DHB matrix in the positive (+ ve) ion mode (0.5 M DHB, 2, 5- dihydroxybenzoic acid, Aldrich 149,357) dissolved in methanol, or an 80% (w/v) acetone solution of 9-AA (9-aminoacridine, Sigma-Aldrich 92,817-IG) as the matrix in the negative (−ve) ion mode. The use of the two matrices is highly recommended as certain lipids are detectable as positive ions (for example PC), while others as negative ions or with very low sensitivity in the positive ion detection mode (for example PS) (Fuchs et al. 2010).

## Results

We recovered and characterised exosomes from CHO-S cell cultures on two days of batch culture, day 3 and 5 when the culture viability was 98.6% and 98% respectively. At these times the viable cell concentration in the cultures was 2.68 × 10^6^ viable cells/ml and 7.10 × 10^6^ viable cells/ml on days 3 and 5 respectively as determined using a Vi-Cell instrument. These timepoints represent (1) mid-late exponential growth and (2) early stationary phase and thus allowed investigation of how the exosome population may change between these timepoints.

### Analysis of isolated CHO cell culture exosomes by dynamic light scattering (DLS) and polydispersity index determination

**Fig. 1:**
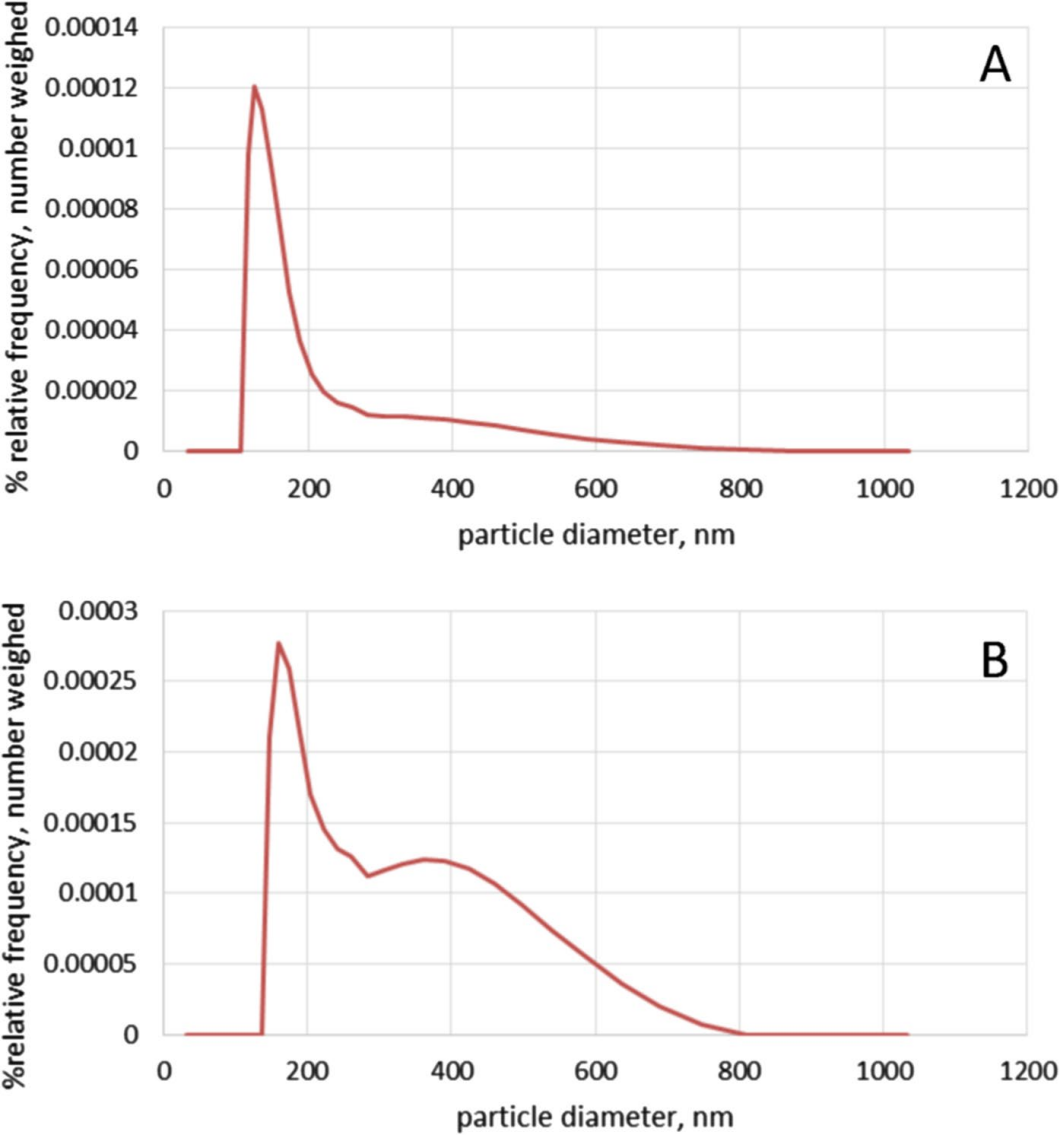
Size distribution of exosome enriched preparations from CHO-S cells harvested on day 3 (**A**) or day 5 (**B**) of batch culture, as determined by DLS analysis

DLS measurements of exosomes harvested at Day 3 and 5 of batch culture were undertaken as described in the methods section and the data is summarised in Fig. 1 and Table 1. All results from DLS measurements were analysed based on the number-weighed relative frequency which is not biased towards the larger vesicles in a dispersion as explained in the Methods section. The average polydispersity index (PDI) was found to be 0.217 on Day 3 and 0.220 on Day 5 of culture (Table 1), in agreement with values in the literature for exosome preparations (Kesimer et al. 2015).

**Table 1:** Polydispersity Index (PDI), range, mean and modal sizes of the exosome preparations from CHO cells from days 3 and 5 of batch culture

Day of Culture	Polydispersity index (PDI)	Size range within which 80% of particles fall (nm)	Mean particle size (nm)	Modal particle size (nm)
3	0.217	126–204	147	130
5	0.220	126–392	220	160

The isolated exosomes consisted of a dispersion of vesicle sizes across that expected for exosomes (Fig. 1). No particles with diameters smaller than 30 nm were taken into account in the analysis as they are not classified as exosomes (observations of 10–20 nm lipid particles in ours and others’ exosome preparations are commonly observed, as noted in Thery et al. 2006). Furthermore, no particles were recorded of diameters greater than around 900 nm in our preparations. Of the vesicles with particle diameter greater than 30 nm, 80% were estimated to be between 126 and 204 nm in diameter with a mean size of 147 nm and modal size of 130 nm on Day 3 of culture (Table 1). On Day 5 of culture, 80% of the vesicles with diameters greater than 30 nm were estimated to be between 126 and 392 nm in diameter with a mean size of 220 nm and modal size of 160 nm (Table 1). The calculations were based on five measurements for each sample and the average mean and modal sizes were calculated based on the use of mean and modal sizes described in Rider et al. (2016).

### Zeta potential determination of isolated exosome preparations

Zeta potential was also determined for the exosome preparations. As for the exosome preparation size analyses by DLS, there was an appreciable difference in the Zeta potential between exosomes harvested on Day 3 or Day 5 of culture (Table 2). Higher Zeta potential values were obtained for samples that had been allowed to equilibrate to room temperature emphasising the importance of a standard operating procedure for taking such measurements.

**Table 2:** Zeta potential of exosomes from CHO–S cells harvested on Days 3 and 5 of batch culture. Exosome samples were stored at − 20 °C prior to analysis and then either thawed and allowed to reach room temperature over 2 h or thawed and then immediately analysed

Day of batch culture	Mean zeta potential (mV)	Standard deviation (mV)
3, room temp equilibrated	− 13.30	± 0.58
3, immediately after thawing	− 11.68	± 0.54
5, room temp equilibrated	− 16.64	± 0.95
5, immediately after thawing	− 13.74	± 0.64

### Electron microscopy analysis of CHO cell exosome preparations

**Fig. 2:**
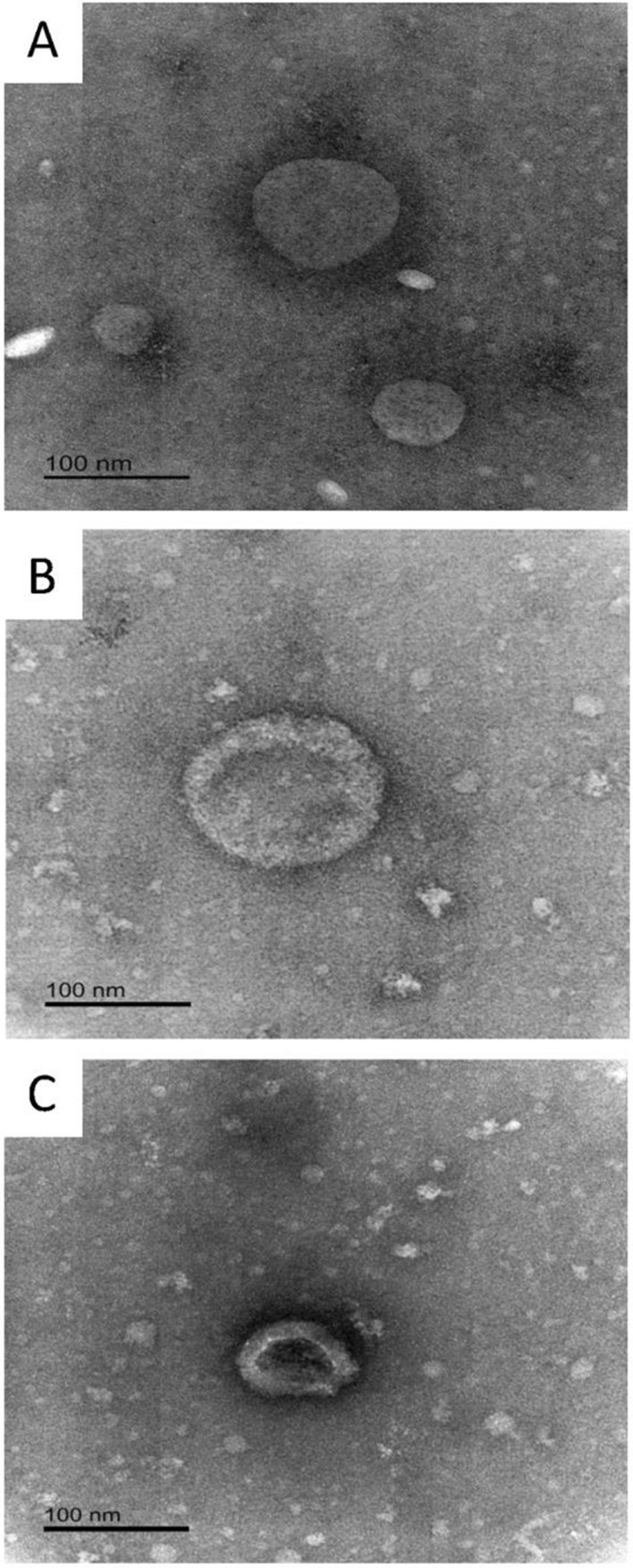
Electron microscopy images of exosome enriched preparations from CHO–S cells harvested at Day 3 (**A**) or Day 5 (**B** and **C**) of batch culture. The bar shows 100 nm

Exosome-sized, characteristic cup-shaped, mostly round or slightly elliptical vesicles were observed under the electron microscope (Fig. 2) in agreement with those reportedly observed by EM for PBP isolated vesicles (Niu et al. 2017; Rider et al. 2016.). The heterogeneity of shapes and sizes observed in samples is reported widely in the literature and has been studied comprehensibly by Zabeo et al. (2017). The sizes observed varied commonly from circa 50 to circa 300 nm, although there was not a systematic or exhaustive analysis of all the vesicles in the EM preparations herein.

### Determination of protein content in exosome preparations

We next estimated the protein content of the isolated exosomes on the different days of culture using the Bradford method to determine protein concentration (Table 3). The amount of protein measured in the whole cell lysates was greater from cells harvested on Day 5 of batch culture compared to Day 3 (Table 3). By contrast, the protein content of the isolated exosomes was higher in Day 3 exosomes compared to Day 5 isolated exosomes (Table 3). Interestingly, lysis of the vesicles resulted in a higher protein content, an indication that intravesicular exosome content constitutes approximately half of the total exosome protein (Table 3).

**Table 3:** Protein content of CHO–S cell lysates and isolated exosomes

Day of Harvest	Protein content (μg/10^6^ cells)		
	Cell lysate	Exosome lysate	Non-lysed exosome preps
3	298.9	5.93	3.08
5	477.2	4.66	2.06

### Determining the presence of specific exosome biomarkers on day 3 and 5 of batch culture in exosome preparations by western blot analysis

We next investigated the presence and enrichment of known exosome biomarkers in CHO cell lysates and exosome preparations by Western Blot using anti-CD63, anti-CD81 and anti-TSG101 antibodies to detect these common exosome markers. This approach is in line with that recommended by Lotvall et al. (2014), that the composition of extracellular vesicles should ideally be compared with that of the parental cells, using at least three exosome markers (Lotvall et al. 2014). Total protein from cell lysates or exosome preparations were resolved under denaturing and reducing or non-reducing conditions. For cell lysates, 6 μg of total protein was loaded onto gels whilst 2.3 and 3.2 μg of protein of the non-lysed and lysed exosomes harvested at Day 3 and 3.2 and 7.0 μg of protein of the non-lysed and lysed exosomes harvested at Day 5 respectively were analysed. As a result, the protein bands visualised in Day 3 exosome preparations were generally much less intense compared with the bands of Day 5 preparations.

**Fig. 3:**
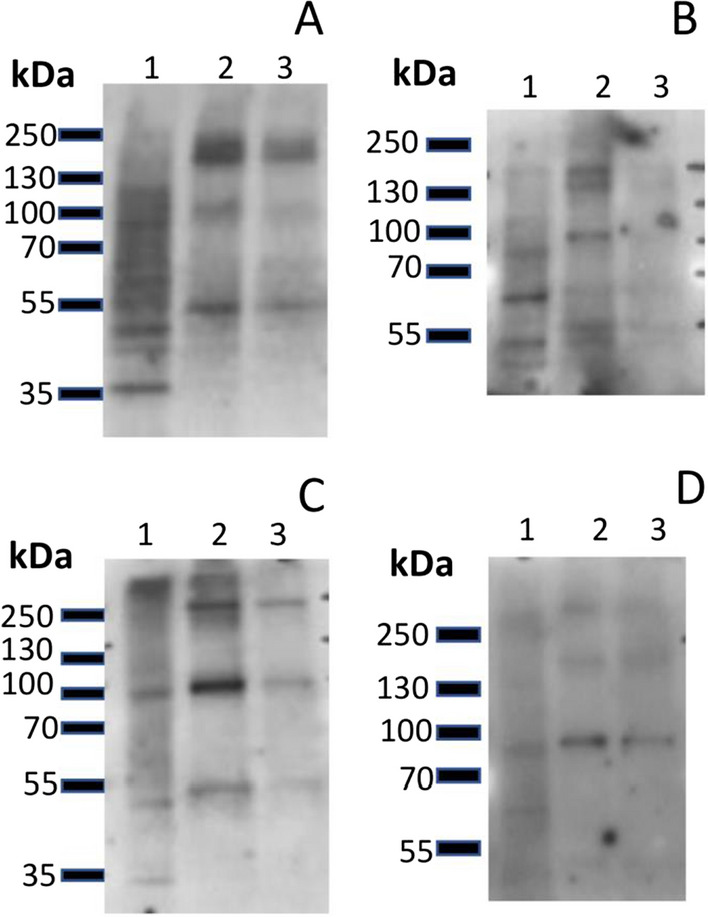
Western blot analysis of the total lysates of CHO–S cells (Lane 1), exosome preparations (Lane 2) and exosome lysates (Lane 3) under reducing (upper panels, **A–B**) and non-reducing (lower panels, C-D) for day 5 samples for the exosome biomarkers CD63 (**A** and **C**) and TSG101 (**B** and **D**)

There was a clear and obvious band when probing for tetraspanin CD63 in the non-lysed, and less so in the lysed, exosome samples under non-reducing conditions compared to the cell lysate for Day 5 samples (panel C, Fig. 3). A more intense signal for CD63 under non-reducing (panel C, Fig. 3), compared to reducing conditions (panel A, Fig. 3) agrees with previous reports when probing for CD63 (Lobb et al. 2015). The apparent molecular weight was approximately 100 kDa with fainter bands observed around 55 and 250 kDa. Whilst the expected size of CD63 is approximately 26 kDa, the observed size is in agreement with other studies. The observed size is thought to be due to glycosylation and polymerisation of the monomeric form (Ferguson et al. 2018; van Niel et al. 2011).

When probing for the presence of the tetraspanin CD81 (expected size 22–26 kDa) a detectable band in the exosomes preparations from at Day 5 under reducing conditions was observed (data not shown), although this was not clear due to the background caused by the long exposure of the film suggesting that there were only low levels of this protein present or that the antibody does not recognise the Chinese hamster protein well. The intensity of the band in the cell lysates was fainter, suggesting an enrichment in the exosome samples.

When probing for the intracellular/intravesicular TSG101 protein (expected size 45 kDa) a number of faint bands around 45 (panel B, Fig. 3) and 100 kDa in the Day 5 cell lysate and Day 5 exosome preparations (panels B and D, Fig. 3), were observed. These results are in agreement with others (van Deun et al. 2014) who reported a faint band for TSG101 in exosomes compared to total cell lysate when exosomes were isolated using the TEI method compared to other isolation methods. Collectively these data confirm the presence of three known exosome markers and provide further evidence that the preparations from the CHO cells were exosomal in nature.

### The RNA content of CHO cell derived exosomes

The concentration of exosomal RNA of three samples from each harvest day are summarised in Table 4. The purity of the eluants for the presence of RNA and the absence of protein and other impurities was high as judged by the 260/280 and 260/230 ratios respectively. The RNA purity confirms findings from Tang et al. (2017) whereby the TEI method of exosome isolation was characterised by the high purity of RNA recovered from such exosome preparations. We also calculated the total amount of RNA in exosomes per 10^6^ cells on the different harvest days (Table 4). Although a range of concentrations was found across different samples, there was a general reduction in the amount of RNA in exosome preparations from Day 5 of culture compared to those from Day 3, in line with the reduction of protein also observed in Day 5 samples compared to Day 3 exosome derived samples. A number of recent reports have now begun to map the RNA content in CHO cell derived exosomes (Busch et al. 2022; Belliveau et al. 2022), describing how exosomes allow the rapid and wide exchange of RNAs between cells in culture. Further expansion of RNA content of CHO cells will not only help in the characterisation of the content but also begin to unravel the importance of exosomes in RNA biology and cell signalling.

**Table 4:** Quantity and Quality of RNA extracted from 3 samples of CHO–S cell derived exosomes on different harvest days (exoRNA refers to exosome RNA concentration)

Harvest day	exoRNA (ng/μl)	A260:280 nm ratio	A260:230 nm ratio	exoRNA ng/10^6^ cells
3	26.9	2.22	0.06	301.1–351.3
3	12.8	3.99	0.03	143.3–167.2
3	23.2	2.34	0.05	259.7–303.0
5	33.1	2.24	0.07	139.9–163.2
5	11.2	4.66	0.03	47.3–55.2
5	15.4	3.19	0.04	65.1–75.9

### Analysis of the lipid content of CHO cell derived exosomes

**Fig. 4:**
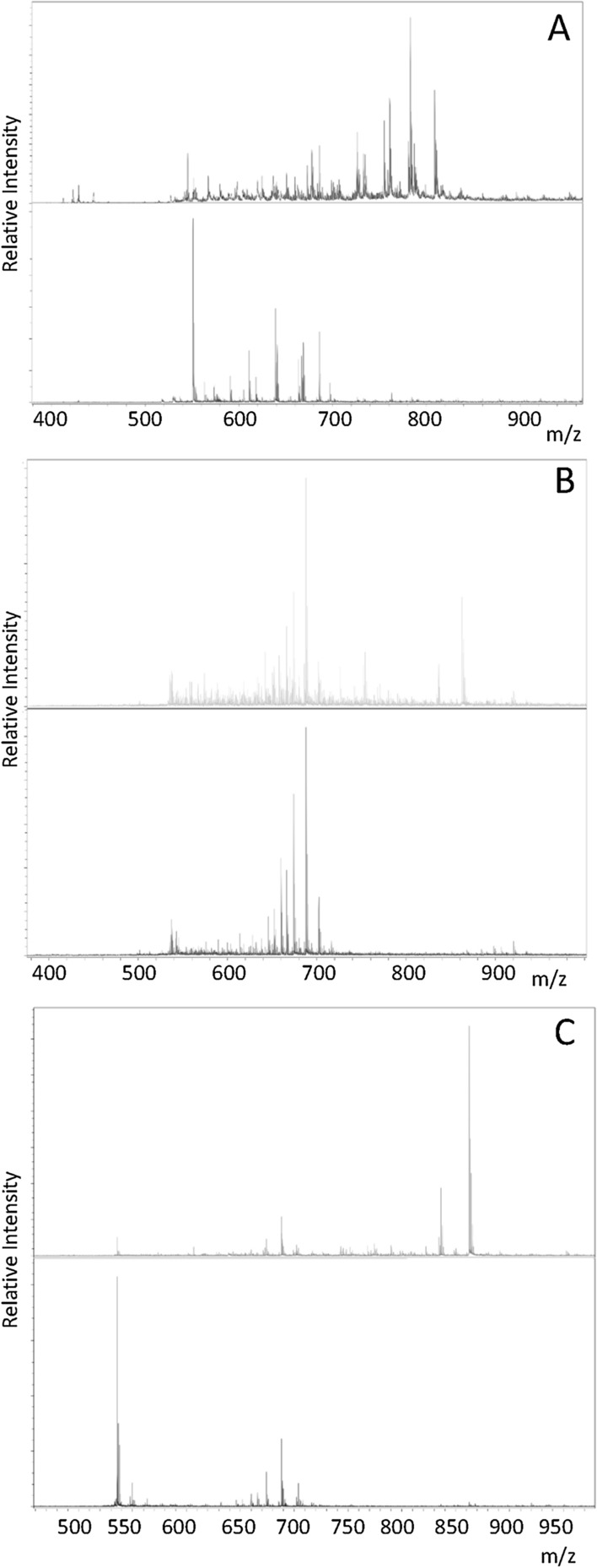
Lipid mass spectrometry profiles profile obtained by MALDI-ToF mass spectrometry of exosomes and cell lysates. The upper spectrum of each panel is of CHO–S cell lipid extracts whilst the lower spectrum is that of CHO cell exosomes from the same harvest day as the cell lysates. **A** Harvest day 3 cell lysate (upper spectrum) and exosome (lower spectrum) lipid profiles using DHB matrix and data collected in the + ve ion mode. **B** Harvest day 3 cell lysate (upper spectrum) and exosome (lower spectrum) lipid profiles using 9-AA matrix and data collected in the −ve ion mode. **C** Harvest day 5 cell lysate (upper spectrum) and exosome (lower spectrum) lipid profiles using 9-AA matrix and data collected in the −ve ion mode

We profiled the lipid content of CHO cells and their derived exosomes from the different batch culture harvest days using MALDI-ToF mass spectrometry, using the standard 1P2S lipid (m/z763) as a normaliser (Fig. 4A–C). We note that the total lipid content of samples was not determined. Although the majority of the lipid ion m/z values observed were detected in both cell and exosome preparations, several were found to be either more abundant in the cell preparations compared to the their exosomes, where these lipids were barely detectable or absent, or the reverse, where the exosomes were enriched in a particular lipid ion compared to the parent cells. Ions with m/z values expected for those of cholesterol (m/z 763, 785) and sphingomyelin (m/z 704) were present in both cells and exosomes, as well as DPPC and DSPC but not DOPC which was low/absent in the exosomes; furthermore, 1P2O was present in the cells but low/absent in the exosomes. Those lipid ion m/z values that were enriched in the parent CHO cells compared to lipids from exosomes derived from those CHO cells where these lipids were either reduced or absent are reported in Table 5. Interestingly, it was observed that lipids with m/z values 551 (Fig. 4A) and 535 (Fig. 4C) were relatively enrichened in the exosomes compared to the parent CHO cells.

**Table 5:** MALDI-ToF mass spectrometry analysis in the − ve and + ve ion mode of lipid constituents of CHO cell cellular and exosome derived lipids. The table reports a comparison between cellular and exosome lipids identifying lipids ions that were considered to be low or absent on exosomes compared to cellular lipid content

Matrix/ion mode	m/z lipid ion low/absent in exosomes	Standard m/z assignment
DHB/ + ve	545.375	
DHB/ + ve	579.920	
DHB/ + ve	677.886	
DHB/ + ve	726.052	
DHB/ + ve	761.138	1P2O
DHB/ + ve	783.073	DOPC
DHB/ + ve	809.127	
9-AA/−ve	726.126	
9-AA/−ve	754.193	
9-AA/−ve	836.439	
9-AA/−ve	863.454	

## Discussion

Here we set out to define some standard measurements of CHO cell derived exosomes for the community. We used DLS to investigate the size of our isolated exosomes and note that the size range of the isolated exosomes were comparable to those of exosomes previously isolated using the same methodology from both HEK and PC3 cells (Ferguson et al. 2018), as well as from human lung cancer cells (Tang et al. 2017). The isolation method investigated here is useful for rapid characterisation of exosomes although density based centrifugation methods are the current gold standard for EV preparations although precipitation based methods such as used here have also been validated (Eguchi et al. 2022). Further, the EM and analyses shown in the results, and the enrichment of classic exosome markers, confirmed the isolation of EVs and thus validated the isolation approach utilised. Nevertheless, we suggest that analysis of other markers of EV preparations and of non-EV contaminants (e.g. LGALS3BP) would be useful additional data to add to our initial characterisation data in the future.

The data in Table 1 and Fig. 1 shows that the DLS analysis revealed differences between the exosomes recovered from Day 3 and Day 5 of batch culture. Firstly, there was increased polydispersity in the exosomes recovered from Day 5; secondly, the size range of the vesicles was greater at Day 5 of batch culture, with larger vesicles present in higher numbers. As a result, the mean size as well as the modal size were larger on Day 5 of batch culture compared to Day 3. These differences may be due to aggregation and/or fusion of secreted vesicles with increased culture time, as well as, and/or the secretion of larger vesicles to the extracellular milieu. Regardless, this difference in properties has implications for the reproducible generation of exosomes from cell culture and should be considered when investigators set harvest day parameters for isolation of CHO cell derived exosomes. We note that investigating only two time points limits the extent to which we could characterise the dynamic nature of the exosomes produced by CHO–S cells across culture, nevertheless at these two key points (exponential growth and stationary phase) clear differences in the nature of the isolated exosomes were observed.

We also measured Zeta potential of the EV preparations. In principle, the higher the absolute value of the Zeta potential, the less likely the dispersed particles are to aggregate. However, it is still uncertain what the significance of Zeta potential values are for biological suspensions such as EV preparations as discussed by Erdbrugger et al. (2016). Nevertheless, as for the exosome preparation size analyses by DLS, there was an appreciable difference in the Zeta potential between exosomes harvested on Day 3 or Day 5 of culture (Table 2). The Zeta potential values observed here are comparable with values obtained from exosomes of HEK and PC3 cells (Ferguson et al. 2018). The only published value for CHO cell exosome preparations is − 10.3 mV, measured in PBS buffer (Han et al. 2018). Interestingly, higher Zeta potential values were obtained for samples that had been allowed to equilibrate to room temperature emphasising the importance of a standard operating procedure for taking such measurements. Furthermore, others have reported that the buffer used to make measurements is important, with others reporting a higher value being obtained when water is used to prepare samples rather than a buffer, which should be considered when values in the literature are compared where measurements have been obtained using different buffer systems (Kesimer et al. 2015).

When the EV preparations were analysed by electron microscopy, the range of sizes observed was in broad agreement with published values (Zhang et al. 2016) and of that found from the DLS analysis. The generally smaller size vesicles observed by EM compared to DLS is likely due to the shrinkage of the vesicles when subjected to the dehydrating technique involved in the preparation protocol for EM analysis, an observation reported by several groups whereby a cup-shaped form is commonly reported to be due to the mode of a samples preparation and fixation (Zhang et al. 2016; Peterson et al. 2015).

When we investigated the protein content of the CHO exosomes, we found that there was more protein in the CHO cell isolated exosomes compared with published values from mast and dendritic cells (300 μg per 10^9^ cells or 0.3 μg per 10^6^ cells, Laulagnier et al. 2004) and from the human prostate cancer cell line PC-3 (0.78 ± 0.13 μg exosome protein per 10^6^ cells, Llorente et al. 2013). The relatively high amount of protein in the CHO cell derived exosome preparations described here compared to other studies may reflect a higher exosome recovery or extraction efficiency and/or protein contamination, both characteristics of the TEI isolation method used here (Tang et al. 2017). Of note, higher exosome protein may be important in exosome-based therapeutics, with suggested doses being 10–100 μg exosome protein/mouse, whereas typically the published yields are less than 1  g exosome protein from 1 ml of culture (Yamashita et al. 2018). Our data shows that the protein in isolated CHO cell exosomes ranged from 8.25 to 33.10 μg exosome protein per ml of culture, suggesting that CHO cells may yield exosomes with higher protein content that could be advantageous for the preparation of therapeutic exosomes. Further, although there was more protein in the cell lysates of the Day 5 cells, there was more protein in the exosome preparation from Day 3 compared to Day 5 when normalised for the same number of cells. This might indicate (a) a higher *rate* of exosome production during the exponential growth phase and/or (b) that the exosome fraction has a higher contamination of intracellular proteins in Day 3 samples, and/or (c) that there is differential packaging or targeting of proteins to exosomes between Day 3 and Day 5 of culture.

We also fingerprinted the lipid profile of the CHO cell derived exosomes. The observation that particular lipids are enriched in cells compared to their exosomes and vice versa are in agreement with the findings of others that have reported differences between cellular and exosomal lipid profiles (Ferguson et al., 2016; Skotland et al. 2017; Llorente et al. 2013; Laulagnier et al. 2004). Although we have not completed an entire lipid analysis here, we report the lipid composition “fingerprint” of CHO–S cell derived exosomes for the first time, an important aspect of lipid analysis and exosome characterisation (Fuchs et al 2010) that can serve as a comparison for future CHO cell exosome studies.

In conclusion, here we provide a characterisation of CHO cell derived exosomes that can be used by others as a comparison and validation of preparation of exosomes derived from this source. We recognise that the exosome isolation approach used here is not currently suitable for large scale exosome isolation and hence for commercial preparation, where centrifugation approaches are likely to be utilised, the exosomes isolated may have different properties. Nevertheless, the analyses reported here provide a detailed fingerprint of CHO cell based exosomes that others in the field can use as a comparator as the field develops. Further work remains to be undertaken in the future to characterise other aspects of exosomes from CHO cells including the identification of additional exosome biomarkers, more complete lipid, protein and RNA analysis of exosomes as well as the biological activity of isolated exosome preparations by devising a suitable assay (Peterson et al. 2015). These studies not only will increase our fundamental understanding of the exosomes from the CHO cells, they will also widen the scope of optimising existing and/or novel biotherapeutic products and applications.

## Data Availability

The datasets generated during and/or analysed during the current study are available from the corresponding author on reasonable request.
